# Multiple origins of resistance-conferring mutations in *Plasmodium vivax dihydrofolate reductase*

**DOI:** 10.1186/1475-2875-7-72

**Published:** 2008-04-28

**Authors:** Vivian N Hawkins, Alyson Auliff, Surendra Kumar Prajapati, Kanchana Rungsihirunrat, Hapuarachchige C Hapuarachchi, Amanda Maestre, Michael T O'Neil, Qin Cheng, Hema Joshi, Kesara Na-Bangchang, Carol Hopkins Sibley

**Affiliations:** 1 Department of Genome Sciences, University of Washington, Seattle WA, USA; 2 Department of Drug Resistance and Diagnostics, Australian Army Medical Institute, Enoggera, Queensland, Australia; 3 National Institute of Malaria Research, Delhi, India; 4 The College of Public Health Sciences, Chulalongkorn University, Bangkok, Thailand; 5 Faculty of Medicine, University of Kelaniya, Ragama, Sri Lanka; 6 Grupo Malaria, Universidad de Antioquia, Medellín, Colombia; 7 Division of Experimental Therapeutics, Walter Reed Army Institute of Research, Silver Spring MD, USA; 8 Faculty of Allied Health Sciences, Thammasat University, Pathumthanee, Thailand

## Abstract

**Background:**

In order to maximize the useful therapeutic life of antimalarial drugs, it is crucial to understand the mechanisms by which parasites resistant to antimalarial drugs are selected and spread in natural populations. Recent work has demonstrated that pyrimethamine-resistance conferring mutations in *Plasmodium falciparum dihydrofolate reductase *(*dhfr*) have arisen rarely *de novo*, but spread widely in Asia and Africa. The origin and spread of mutations in *Plasmodium vivax dhfr *were assessed by constructing haplotypes based on sequencing *dhfr *and its flanking regions.

**Methods:**

The *P. vivax dhfr *coding region, 792 bp upstream and 683 bp downstream were amplified and sequenced from 137 contemporary patient isolates from Colombia, India, Indonesia, Papua New Guinea, Sri Lanka, Thailand, and Vanuatu. A repeat motif located 2.6 kb upstream of *dhfr *was also sequenced from 75 of 137 patient isolates, and mutational relationships among the haplotypes were visualized using the programme Network.

**Results:**

Synonymous and non-synonymous single nucleotide polymorphisms (SNPs) within the *dhfr *coding region were identified, as was the well-documented in-frame insertion/deletion (indel). SNPs were also identified upstream and downstream of *dhfr*, with an indel and a highly polymorphic repeat region identified upstream of *dhfr*. The regions flanking *dhfr *were highly variable. The double mutant (58R/117N) *dhfr *allele has evolved from several origins, because the 58R is encoded by at least 3 different codons. The triple (58R/61M/117T) and quadruple (57L/61M/117T/173F, 57I/58R/61M/117T and 57L/58R/61M/117T) mutant alleles had at least three independent origins in Thailand, Indonesia, and Papua New Guinea/Vanuatu.

**Conclusion:**

It was found that the *P. vivax dhfr *coding region and its flanking intergenic regions are highly polymorphic and that mutations in *P. vivax dhfr *that confer antifolate resistance have arisen several times in the Asian region. This contrasts sharply with the selective sweep of rare antifolate resistant alleles observed in the *P. falciparum *populations in Asia and Africa. The finding of multiple origins of resistance-conferring mutations has important implications for drug policy.

## Background

In order to maximize the useful therapeutic life of antimalarial drugs, it is crucial to understand the mechanisms by which parasites resistant to antimalarial drugs are selected and subsequently spread in natural populations. This is a major issue in *Plasmodium falciparum*, where resistance to two safe, inexpensive drugs, chloroquine and sulphadoxine-pyrimethamine, has spread widely in endemic areas (for review see [1,2]). Chloroquine has been the drug of choice for *Plasmodium vivax *for decades, but resistance has been reported in several areas of south-east Asia, and the spread of these resistant strains has serious implications for public health [3]. While sulphadoxine-pyrimethamine is not generally recommended as a treatment for *P. vivax*, in many regions *P. vivax *is sympatric with *P. falciparum *[4-6]. Due to presumptive treatment for *P. falciparum*, misdiagnosis or mixed-species infection, *P. vivax *populations are sometimes inadvertently exposed to sulphadoxine-pyrimethamine pressure. Thus, study of the resistance to pyrimethamine in *P. vivax *can be a very useful tool to understand the evolution of resistance in these parasite populations.

Point mutations in the gene that encodes dihydrofolate reductase (DHFR) confer resistance to pyrimethamine in both *P. vivax *and *P. falciparum*, and mutant alleles have been identified in most endemic regions (for review see [1,7-9]). In *P. falciparum*, non-synonymous single nucleotide polymorphisms (SNPs) that alter the amino acids within the active site of the enzyme at residues 50, 51, 59, 108 and 164 have been demonstrated both *in vitro *and *in vivo *to confer resistance to pyrimethamine [1]. Studies of the *P. vivax dhfr *gene have associated changes at amino acids 49, 57, 58, 61, 117 and 173 with pyrimethamine resistance (for review see [8]). Not surprisingly, most of these amino acid changes are also located at similar positions, as compared to *P. falciparum*, within the active site for the *P. vivax *enzyme. A recent study conducted in India has found that mutations in *P. vivax dhfr *and *P. falciparum dhfr *are correlated, and that resistance-conferring mutations in *P. vivax dhfr *are more common in areas where *P. falciparum *is the major *Plasmodium *species and where sulphadoxine-pyrimethamine usage is highest. These findings strongly suggest that sulphadoxine-pyrimethamine used to treat *P. falciparum *exerts substantial selective pressure on *P. vivax *populations, providing a selective advantage to parasites bearing resistance-conferring mutations in *P. vivax dhfr *[10].

Despite these similarities between the two systems, there are some striking differences. Most important, the *P. vivax dhfr *gene is highly polymorphic. Over 20 different alleles of the *dhfr *coding region have been identified from a limited geographic sampling and insertions/deletions (indels) and synonymous and non-synonymous changes are common (for review see [8]). In contrast, only a handful of *P. falciparum dhfr *alleles have been identified from far more extensive surveillance, and insertions/deletions and non-synonymous changes are exceedingly rare (for review see [7]). Thus, the mutation set seen in *P. vivax dhfr *is substantially more diverse than that seen in *P. falciparum dhfr*. It is important to acknowledge that due to difficulties with *in vitro *culture of *P. vivax *our knowledge of the impact of mutations in *dhfr *on resistance to pyrimethamine has been derived from yeast and bacterial transfection systems and from a limited number of trials in human subjects (for review see [7]). This means that definition of true *in vivo *and *in vitro *resistance-conferring *dhfr *alleles is somewhat challenging and controversial.

Several groups have assessed the origin and spread of resistance-conferring mutations in *P. falciparum dhfr *by assessing microsatellites flanking the gene [11-17]. Remarkably, the highly resistant triple-mutant *P. falciparum dhfr *allele in Africa shares a common origin with *dhfr *alleles bearing two to four mutations in Southeast Asia [17]. In contrast, these loci show high levels of variation around sensitive *dhfr *alleles. Thus, mutations that confer pyrimethamine resistance have arisen *de novo *extremely rarely in *P. falciparum *and most resistant alleles in Asian and African populations are identical by descent, indicative of a selective sweep of this allele in these populations.

However, recent work has suggested that the common triple mutant allele *of dhfr *may have arisen independently in Kenyan populations of *P. falciparum *[12]. Furthermore, it has been found in South America that a triple mutant allele of *P. falciparum dhfr *arose on a haplotype different from that observed in Southeast Asia and Africa [11].

Recently one group has attempted to examine the evolution of resistance-conferring mutations in *P. vivax dhfr *by assessing microsatellites positioned 38.83 and 230.64 kb upstream of *dhfr *and 6.15 kb and 283.28 kb downstream [18]. These microsatellite loci were found to be highly polymorphic, with expected heterozygosity ranging from 0.50 to 0.82. However, there was no association between mutations in *P. vivax dhfr *and the flanking microsatellites. It may be speculated that this lack of association may have been due, in part, to the great distance between the assessed microsatellites and the gene. For these microsatellites to have hitchhiked along with the *dhfr *gene selection would have had to have been extremely strong and quite recent, with little recombination occurring to break down associations and little mutation of microsatellites [19]. Given these stipulations, it may not be surprising that there was no association between the flanking microsatellites and *P. vivax dhfr*.

In any case, not all drug pressure has produced dramatic selective sweeps of the *P. falciparum *population. The point mutations in the *cytochrome b1 *gene that confer resistance to the atovaquone component of Malarone^® ^have occurred repeatedly and independently [20]. The increased copy number that confers resistance to mefloquine also appears to have been selected in multiple independent sites, even within the small area of Southeast Asia where the drug has been intensively used [14,21]. Thus, the selective sweep paradigm may not apply to resistance to all antimalarial drugs even in *P. falciparum*.

Given the differences in diversity of *P. vivax dhfr *alleles as compared to *P. falciparum*, it cannot be assumed that *P. vivax *will follow the pattern of selective sweep generally observed for *P. falciparum dhfr*. To address this question, in this paper the origin and spread of resistance-conferring mutations in *P. vivax dhfr *are assessed.

## Materials and methods

### Orientation to *Plasmodiumvivax dhfr-ts*

The *P. vivax *genome was recently sequenced by The Institute for Genomic Research (TIGR) [22]. 3,306 bp lie between *P. vivax dhfr-ts *and the nearest upstream ORF, and 880 bp lie between *P. vivax dhfr-ts *and the nearest downstream ORF; there are no introns.

### Sample sources and DNA extraction

Genomic DNA was extracted from filter paper blotted with the blood of patients infected with *P. vivax *(Qiagen, Rockville MD). *P. vivax dhfr *and 792 bp upstream/683 bp downstream were amplified and sequenced from 137 contemporary patient isolates (Colombia, *n *= 9; India, *n *= 24; Indonesia, *n *= 43; Papua New Guinea, n = 7; Sri Lanka, *n *= 16; Thailand, *n *= 29; Vanuatu, *n *= 9) and from the MR4 repository, we assessed samples from Panama (1966), Nicaragua (pre-1986), Pakchong (Thailand, 1972), and ONG (Vietnamese refugee who had spent time in Indonesia, collected 1980) [23,24]. Amplification of Indian isolates was performed within India, and amplicons were sent to the United States for sequencing.

The region further upstream of *P. vivax dhfr*, to 3,258 bp upstream, was amplified and sequenced from a subset of 75 of the 137 patient isolates (Colombia, *n *= 7; Indonesia, *n *= 24; Sri Lanka, *n *= 13; Papua New Guinea, *n *= 7; Thailand, *n *= 17; Vanuatu, *n *= 7) and from the 4 MR4 isolates described above. In addition, *ts *was sequenced from 19 patient isolates (Colombia, *n *= 8; Indonesia, *n *= 4; Thailand, *n *= 7).

The Colombian samples were collected from Urabá, on the western coast of Colombia, in 2005. The Indonesian samples are derived from two locations, Papua (*n *= 29) in 1996 to 1999 and Java (*n *= 14) in 2000, which are separated by approximately 3,200 km. The Thai samples are derived from the Thai/Cambodian border (*n *= 6) and from the Thai/Myanmar border (*n *= 23) in 2005. The Sri Lankan samples are derived from the north of the island (*n *= 12), and the south (*n *= 3) in 2003; in addition, there is one Sri Lankan isolate whose geographic origin is unknown. The Indian isolates are derived from Nadiad, Gujarat (*n *= 6), Delhi (*n *= 11), Chennai, Tamilnadu (*n *= 4), and Goa (*n *= 3) and were collected during 2003 to 2005. The Vanuatu isolates are from Malo Island in 2005 (*n *= 9). The Papua New Guinea isolates are from East New Britain Province in 1989 (*n *= 2), from an unknown location passaged through *Aotus *monkey, collected 1989 (*n *= 1), and from Lae in 2006 (*n *= 4).

All isolates were derived from studies that had been approved by the appropriate local Institutional Review Board and these anonymous samples are in the exempt category of the University of Washington Human Subjects Review Board.

### Amplification *of Pvdhfr *and flanking region (Product A: 792 bp upstream to 683 bp downstream)

A nested PCR protocol was used. Primer sequences are given in Table 1. For the first-round amplification of *Pvdhfr *and flanking, 0.2 μL FailSafe enzyme (Epicentre Technologies, Madison WI), 1 μL each 20 μM S1048 and S1049, 2 μL genomic DNA, 10 μL FailSafe buffer E, and water were combined to a total volume of 20 μL. The PCR protocol uses modified hotstart, in which reactions are transferred to the PCR machine only when the machine has reached 94C. For both first and second round amplifications the same protocol was used: 94C, 3 min, followed by 5 cycles of 94C: 30 sec, 67C: 45 sec, 72C: 3 min 25 sec; 5 cycles of 94C: 30 sec, 65C: 45 sec, 72C: 3 min 25 sec; 5 cycles of 94C: 30 sec, 63C, 45 sec, 72C, 3 min 25 sec; 3 cycles of 94C: 30 sec, 61C: 45 sec, 72C, 3 min 25 sec; 3 cycles of 94C: 30 sec, 59C: 45 sec, 72C: 3 min 25 sec; 3 cycles of 94C: 30 sec, 57C: 45 sec, 72C, 3 min 25 sec; 5 cycles of 94C: 30 sec, 55C: 45 sec, 72C, 3 min 25 sec, with a final extension at 72C: 5 min.

**Table 1:** Primers for amplification and sequencing of *Pvdhfr-ts *and flanking, using PCR product A (amplifies *P. vivax dhfr-ts *and 792 bp upstream, 683 bp downstream). All sequences are written 5' to 3'

Primer name	Use	Sequence
S1048	PCR A 1st nested	TGA ACA GCC AAG CGA ATA GGT AAA A
S1049	PCR A 1st nested	ATT TGA AGG TTA AAA TGG GGT GAC G
S1105	PCR A 2nd nested/sequencing	ATG CAC ACA TCT TCG CTC TC
S1106	PCR A 2nd nested/sequencing	TCT TTT TCA CGT TCG CTG TG
S303	Sequencing	ATG GAG GAC CTT TCA GAT GTA TT
S1090	Sequencing	AAA AAA ATA TGC ACT CCC CAT TT
S1092	Sequencing	ACT TTT ATA GCT AGC TAG CGA AGT GTT
S1166	Sequencing	TTT TCT TCG CGG CGA CAA
S1088	Sequencing	CAG TTA TAT GCA CAC ATC TTC GC
S1095	Sequencing	ACT GCG GAC AGC GCT TCG
S1096	Sequencing	TGA AGA TTA AGC AGC ACC CAG
S1098	Sequencing	ATG GCT TTA CCT CCT TGT CA

For the second-round amplification of *Pvdhfr *and flanking, 1 μL FailSafe enzyme (Epicentre Technologies, Madison WI), 5 μL each 20 μM S1105 and S1106, 10 μL 1^st ^nested product (used without any clean-up), 50 μL FailSafe buffer E, and water were combined to a total volume of 100 μL. Products were cleaned with the GeneClean kit (Qbiogene, Solon OH), following manufacturer's instructions; products were quantified by running 5 μL GeneCleaned product on a 0.7% ethidium bromide-stained agarose gel.

### Amplification further upstream of *Plasmodium vivax dhfr *(Product B: 420 bp upstream to 3258 bp upstream)

A nested PCR protocol was used. Primer sequences are given in Table 2. For the first-round amplification of the region further upstream of *Pvdhfr*, 0.2 μL FailSafe enzyme (Epicentre Technologies, Madison WI), 1 μL each 20 μM S1238 and S1239, 2 μL genomic DNA, 10 μL FailSafe buffer E, and water were combined to a total volume of 20 μL. The PCR protocol uses the same modified hotstart procedure as above. For both first and second round amplifications the same protocol was used: 94C, 3 min, followed by 5 cycles of 94C: 30 sec, 67C: 45 sec, 72C: 3 min; 5 cycles of 94C: 30 sec, 65C: 45 sec, 72C: 3 min; 5 cycles of 94C: 30 sec, 63C, 45 sec, 72C, 3 min; 3 cycles of 94C: 30 sec, 61C: 45 sec, 72C, 3 min; 3 cycles of 94C: 30 sec, 59C: 45 sec, 72C: 3 min; 3 cycles of 94C: 30 sec, 57C: 45 sec, 72C, 3 min; 5 cycles of 94C: 30 sec, 55C: 45 sec, 72C, 3 min, with a final extension at 72C: 5 min.

**Table 2:** Primers for amplification and sequencing of *Pvdhfr-ts *and flanking, using PCR product B (amplifies the region from -3258 bp upstream to -420 bp upstream of *P. vivax dhfr-ts*). All sequences are written 5' to 3'

Primer name	Use	Sequence
S1238	PCR 1^st ^nested	ATC AAG GAA GGC AGA CTC CA
S1239	PCR 1^st ^nested	AGC GTA CTG CCG TCG AAA TA
S1259	PCR 2^nd ^nested, sequencing	GCC TGG TTA CTT TTG GTG GA
S1240	PCR 2^nd ^nested, sequencing	AAA AAC TGA GGC CAC ATT CG
S1241	sequencing	ACT TCT CTC CTG GGC AGA CTT
S1242	sequencing	GAG AGT TGG TAA TGC GGG G
S1243	sequencing	CAT GGC TGG GGA AGG CTC
S1244	sequencing	CCC TTA ACC CGC ATG CAC
S1245	sequencing	CTC CCC CCA TGG GAC AAA AA
S1246	sequencing	TTT GAT TTG ATT TGA TTT GAT TTG A
S1247	sequencing	ATG CCA CAG GGA AGT TAC AG
S1248	sequencing	CAT TTT TCA CAT TTT GGA AA
S1249	sequencing	CCT CGC GCG GGG GGG AAA
S1250	sequencing	TGC TGC AAT GCA AGT GGG T

For the second-round amplification of *Pvdhfr *and flanking, 1 uL FailSafe enzyme (Epicentre Technologies, Madison WI), 5 μL each 20 μM S1259 and S1240, 10 μL 1^st ^nested product (used without any clean-up), 50 μL FailSafe buffer E, and water were combined to a total volume of 100 μL. PCR products were cleaned and quantified as above.

### Sequencing

Sequencing was performed using an ABI capillary sequencer (AppliedBiosystems Foster City CA), and double coverage with independent primers was used (Tables 1 and 2). Sequences were aligned and analysed with Sequencher (Gene Codes, Ann Arbor MI). Insertions and deletions were hand verified. Sequences were aligned with the Sal I reference sequence recently sequenced by TIGR [22].

### Data analysis

The freely available programme Network was used to generate median joining networks [25]. To aid in generation of Network diagrams, sequences were aligned with the commercially available programme DNA Alignment version 1.1.3.0 (Fluxus Engineering, Suffolk England). Briefly, the median joining network allows visualization of the mutational paths that may have led to the observed data; haplotypes are linked based on the assumption that mutations are more likely to derive from a more frequent haplotype and proceed to a less frequent haplotype. In interpreting network diagrams, circles represent haplotypes; the diameter of the circle is proportional to the number of isolates represented. The length of lines linking haplotypes is proportional to the number of mutational steps separating haplotypes. Nodes (black circles) represent hypothetical ancestral haplotypes linking the presently extant haplotypes, or may represent haplotypes that, though presently extant, were not sampled.

The Network diagram is especially helpful when trying to determine whether multiple haplotypes of a given *dhfr *allele are the result of independent origins or of mutations accumulating on an already established haplotype. In the case where multiple haplotypes of a given *dhfr *allele are present as a result of independent mutations, the haplotypes will appear far from one another on the Network diagram, separated by a large number of mutational steps. In the case where multiple haplotypes of a given *dhfr *allele are present as a result of mutations accruing on one genetic background (i.e. there is one origin of the allele of interest, with additional polymorphisms accumulating over time), the haplotypes will cluster, and will be separated by a limited number of mutational steps.

For the presented Network diagram, 139 sequences, from 792 bp upstream to 683 bp downstream (PCR product A), were included. All 4 MR4 isolates were included, as well as 135 of the 137 patient isolates, for a total of 139 sequences. The two patient isolates not included in the presented Network diagram are described below. The Network programme was designed to compare sequences that differ only by SNPs, and a multi-nucleotide indel region was identified upstream of *dhfr*, and another multi-nucleotide indel was identified within the *dhfr *coding region. To accommodate these differences in the programme, both of these indels were coded as an insertion of a single nucleotide relative to the wildtype sequence, and presented in the Network diagram in that way. There were five indel types for the upstream indel, and four indel types for the indel within *dhfr*. Because one upstream indel type was carried by a single parasite (Sri Lankan isolate with synonymous mutation in codon 69 and upstream indel type E), this isolate was dropped from the Network diagram. Thus, only four upstream indel types were considered. For entering sequences into Network, the actual sequence of the inserted or deleted region was recoded as wildtype; all other polymorphisms were left as is. Sequences with neither an upstream indel nor an indel within *dhfr *were left as is, while those with an upstream indel had the nucleotide A, G, T or C, to represent upstream indels A, B, C, and D, respectively, appended to the end of their upstream sequence. Sequences with an in-frame indel in *dhfr *had the nucleotide A, G, T or C, to represent indels 1, 2, 3 and 4, respectively, appended to the beginning of their *dhfr *sequence. Sequences with both an upstream and in-frame indel had one nucleotide appended to the end of their upstream sequence, and one nucleotide appended to the beginning of their *dhfr *sequence as described above. This coding system was used because indels are likely to be the result of the appearance or disappearance of large segments of DNA as a unit, not by addition or subtraction of one nucleotide at a time. By entering sequence data with indels coded as the insertion of a single nucleotide relative to the wildtype sequence, the Network software more accurately portrays the relationships between alleles.

In addition, for the presented Network diagram, one Indonesian quadruple mutant (the isolate wildtype at nucleotide 581 upstream of *dhfr*) was excluded. This isolate was excluded from the Network diagram because its inclusion created a spurious link between the Papua New Guinean quadruple mutants and the remainder of the Indonesian quadruple mutants. With this Indonesian quadruple mutant included in the dataset, the generated Network diagram is identical to that presented herein except that there is a bridge between the Papua New Guinean quadruple mutants and the Indonesian quadruple mutants.

## Results and Discussion

### Plasmodium vivax dhfr-ts

**Figure 1:**
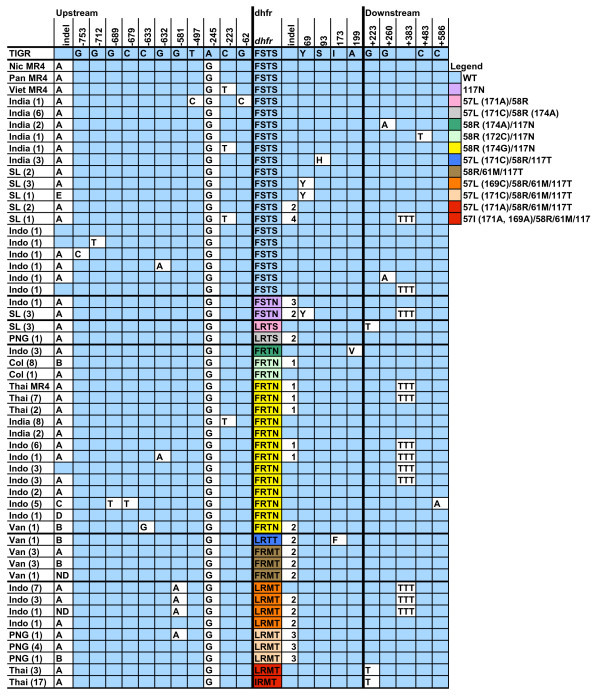
**The *dhfr *and flanking region haplotypes of contemporary global isolates (*n *= 120) and MR4 samples (*n *= 4)**, with each row indicating one distinct haplotype and the number of isolates with that haplotype. Only polymorphic positions are included; blue cells indicate identity with the TIGR reference. The figure is divided vertically into thirds: the region upstream of *P. vivax dhfr*, *dhfr *itself, and the region downstream of *dhfr*. Numerals in the first row indicate the position of the polymorphic nucleotide relative to *dhfr *('-' refers to positions upstream of *dhfr*, '+' refers to positions downstream of *dhfr*). For the upstream indel region, a: deletion of bases [-710, -677], b: deletion of bases [-729, -679], c: deletion of bases [-710, -694], d: insertion of 17 bases after nucleotide -675 (act ggg ggg aaa tgc tc), e: deletion of bases [-746, -696]. Within dhfr, the 4 letters within a single column refer to the amino acids at positions 57, 58, 61 and 117. For the indel within the *dhfr *coding region, 1: deletion of codons 98–103, 2: deletion of codons 93–98, 3: insertion of 18 nucleotides between wildtype codons 98 and 99 (agc ggt ggt gac aac aca), 4: insertion of 18 bp between codons 99 and 100 (tgg tga caa cac aag cgg). Within *dhfr*, the numerals 69, 93 and 199 refer to synonymous or non-synonymous changes at those codons. If the nucleotide mutation yielding 58R is not indicated, the change is 174G. The Thai 57L/58R/61M/117T alleles carry A at nucleotide 171, while the Thai 57I/58R/61M/117T alleles carry A at nucleotide 171 and A at nucleotide 169. Countries are abbreviated as follows: Colombia (Col), Indonesia (Indo), Nicaragua (Nic), Panama (Pan), Papua New Guinea (PNG), Sri Lanka (SL), Thailand (Thai), Vanuatu (Van), Vietnam (Viet).

Previous studies of the coding region of *P. vivax dhfr *have demonstrated considerable genetic diversity among the many pyrimethamine-resistant alleles (for review see [8]). This observation suggested that the diversity might originate from multiple origins, rather than the very few foci observed among pyrimethamine-resistant alleles of *P. falciparum dhfr *[11-17]. Because of the relative scarcity of microsatellites in the *P. vivax *genome, we chose to sequence *P. vivax dhfr *and the flanking intergenic region, and to construct haplotypes based on sequence data. *Pvdhfr *and its flanking regions (792 bp upstream to 683 bp downstream) were sequenced from a geographically diverse set of isolates (Figure 1). *Pvdhfr *is encoded by the N-terminal domain of the bifunctional gene that also encodes thymidylate synthase [26]. At the outset of the study, the entire *dhfr-ts *gene was sequenced. No polymorphisms were identified in the *ts *coding region or linker, sequenced from a subset of diverse isolates (*n *= 19), and so DNA sequences of these domains were not further studied. The diversity within and surrounding the *dhfr-ts *locus provided the polymorphisms needed to begin to infer whether the pyrimethamine-resistant alleles of *P. vivax *also show a signature of a selective sweep.

In order to present a logical framework for this complex dataset, the haplotypes of isolates wildtype at *dhfr *codons 57, 58, 61 and 117 will be presented first, followed by isolates with progressively more mutated *dhfr *alleles. Non-synonymous changes in these four codons will be examined first because they have been shown to specify key amino acids in pyrimethamine-resistance. Of the total 141 isolates examined, 32 isolates carried an allele wildtype at these positions. Among these isolates the *dhfr *coding region contained both synonymous and non-synonymous mutations as well as four distinct in-frame indels, abbreviated 1 through 4 in Figure 1, and described in the figure legend. The flanking regions of these alleles carried a variety of single nucleotide polymorphisms (SNPs). All isolates assessed in this study differed from the TIGR reference by the substitution of a G for an A at nucleotide 245 upstream of *P. vivax dhfr*. A highly polymorphic indel region upstream of *dhfr*, abbreviated as A through E in Figure 1, was also identified. All together there were 14 extended haplotypes carried by the 32 isolates wildtype at codons 57, 58, 61 and 117.

The alleles mutant at codons 57, 58, 61 and 117 are arranged in Figure 1 according to increasing numbers of non-synonymous SNPs in the coding region. Four isolates carried the 117N allele, with two distinct haplotypes. Four isolates carried an allele that encoded 57L/58R, but two different nucleotide substitutions encoded the leucine at codon 57. The Sri Lankan isolates bearing the 57L/58R allele had an adenine at nucleotide 171, while the 57L/58R Papua New Guinean isolate carried a cytosine at that position. Based on the differences in the *dhfr *coding region, including the in-frame indel, and the flanking intergenic region, it is clear that in this dataset we have identified two origins of the 57L/58R allele.

Previous work had documented a double mutant allele, 58R/117N, that is widely disbursed in South Asia (for review see [8]) and 54 of the isolates in the present study carried an allele that encoded this amino acid combination. Thirteen haplotypes were observed in this group, including alleles with 3 different nucleotide changes that all specify an arginine at codon 58. Forty two of these isolates specified the 58R with a guanine at nucleotide 174; these came from a variety of locations in India, Java and Papua Indonesia, the Thai/Cambodian border and the Thai/Myanmar border, and one isolate from Vanuatu. Three isolates from Papua Indonesia had an adenine at nucleotide 174, and the isolates from Colombia were unique with a cytosine at nucleotide 172. This convergent evolution is likely to reflect selection pressure of pyrimethamine on these isolates. Based on these *Pvdhfr *coding region differences and on the flanking polymorphisms, the overall diversity of *Pvdhfr *is considerably higher than that observed in double mutant alleles of *Pfdhfr*.

The major goal of this study was to examine whether highly pyrimethamine-resistant alleles of *Pvdhfr *have arisen in many locations or, like *P. falciparum*, appear to have a limited number of origins. To answer this question, the haplotype pattern of the highly mutant triple and quadruple mutant *Pvdhfr *alleles are of most interest. Seven isolates from Vanuatu that carried a triple mutant allele, 58R/61M/117T, were identified. These isolates had identical *dhfr *coding regions, but differed in the upstream indel region. One isolate from Vanuatu carried a novel quadruple mutant 57L/58R/117T/173F allele, with the 57 leucine encoded by a cytosine at nucleotide 171, but with a haplotype in the flanking regions similar to the 58R/61M/117T triple mutants.

Isolates that encoded the 57L/58R/61M/117T or 57I/58R/61M/117T allele were identified from three locations: Indonesian Papua, Papua New Guinea and western Thailand. Based on differences in both coding and flanking regions, there appear to be several distinct origins of these quadruple mutant alleles, as well. Eleven of 12 Indonesian quadruple mutants share identical upstream and downstream flanking sequences: a G to A mutation at nucleotide 581 upstream of *dhfr *and an insertion of three thymines at nucleotide 383 downstream of *dhfr*. All Indonesian quadruple mutants carried a cytosine at nucleotide 169; four of these isolates also have an in-frame indel within *dhfr *(listed as 2 in Figure 1). One Indonesian quadruple mutant isolate had a unique haplotype and carried neither the SNP at 581 upstream nor the TTT insertion downstream of *dhfr*.

The 57L/58R/61M/117T allele was observed in 3 Thai isolates; the 57 leucine was encoded by an adenine at nucleotide 171. The remaining 17 Thai quadruple mutants carried the 57I/58R/61M/117T allele, with adenines at nucleotides 169 and 171. All of these Thai quadruple mutant isolates shared a common haplotype different from that characteristic of the Indonesian quadruple mutant isolates.

Finally, the Papua New Guinean isolates with a quadruple mutant allele have a cytosine at nucleotide 171, yielding the 57L. All six isolates have similar flanking haplotypes, but differ in that one PNG quadruple mutant carries upstream indel B while the others carry upstream indel A. In addition, one isolate carries a SNP at nucleotide 581 upstream. Quadruple mutant alleles from Papua New Guinea dating from 1989 and 2006 were assessed; isolates from each time-point contained nearly identical flanking regions, suggesting that this particular quadruple mutant haplotype was well established in 1989 and continued to be prevalent through 2006.

**Figure 2:**
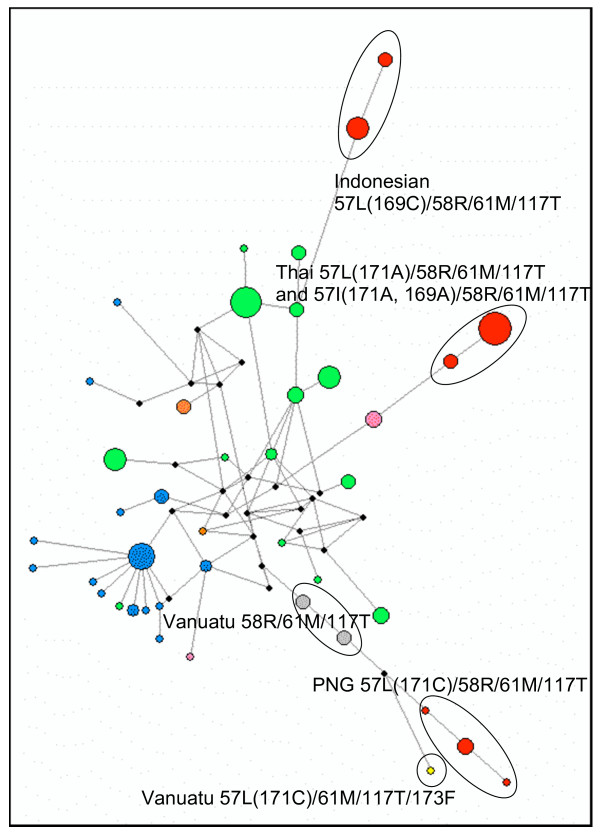
**Network diagram of *P. vivax dhfr *and flanking**. Note that only haplotypes with more than one isolate are included in the diagram. Coloured circles represent *dhfr *and flanking haplotypes; lines represent mutational steps connecting haplotypes, and black nodes represent hypothetical ancestral haplotypes or haplotypes present in the population but not sampled. Colour coding is as follows: wildtype, blue; 58R/117N, green; 117N, orange; 57L/58R, pink; 57L/61M/117T/173F, yellow; 58R/61M/117T, grey; 57I(L)/58R/61M/1117T, red. Note the wide separation between the Thai and Indonesian quadruple mutant alleles, and the triple and quadruple mutants from PNG and Vanuatu.

To better visualize the relationships among these varied alleles of *Pvdhfr*, the data set was analysed using the Network programme described in detail in the Methods section. The diagram in Figure 2 summarizes this analysis. Inspection of the diagram allows several inferences to be drawn. First, the wild type alleles (in blue) mostly cluster together, reflecting the various SNPs and common indels by which they differ. The wide spread 58R/117N double mutant alleles (in green) show a more dispersed pattern, suggesting that they have several origins. This is underlined by the three different nucleotide changes that all convergently encode the 58R amino acid. The triple mutant allele, 58R/61M/117T (in grey) was observed only in Vanuatu, and these cluster together, along with the Vanuatu 57L/61M/117T/173F allele (in yellow).

The patterns of relatedness among the quadruple mutants are most interesting. There are three clusters, reflecting apparent origins of the allele that encodes the 57L(I)/58R/61M/117T enzyme in Indonesia, Thailand and Papua New Guinea. The Thai haplotypes differ only in the non-synonymous SNP that changes codon 57 from leucine to isoleucine, and cluster tightly as expected. The quadruple mutant alleles from PNG also cluster together; these isolates also cluster with the triple and quadruple mutants from Vanuatu. The Indonesian quadruple mutant isolates cluster together clearly separate from the Thai and PNG/Vanuatu groups. These data support strongly the inference that highly pyrimethamine resistant alleles of *Pvdhfr *have arisen in the Southeast Asian region independently. In the present sample set there are three distinct origins of isolates bearing the highly pyrimethamine-resistance conferring 117T allele, with origins in Thailand, Indonesia, and PNG/Vanuatu.

**Figure 3:**
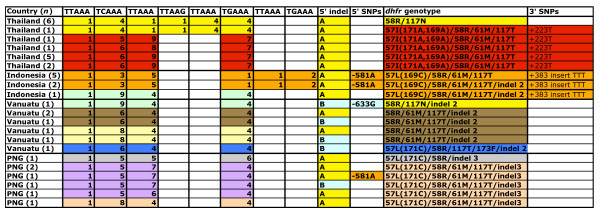
**5' repeat motif, upstream flanking region, *dhfr *genotype and downstream flanking region of selected isolates**. Numbers in columns 2 through 9 indicate the number of repeats of a given type, with the first repeat beginning 2,608 bp upstream of *dhfr *per the reference sequence. Colours in the *dhfr *genotype column reflect *dhfr *genotype, as in Figure 3. Colours upstream and downstream of *dhfr *reflect similarity of flanking regions. The 5' indel and in-frame indel within *dhfr *are coded as in Figure 3. All isolates bear the 174G mutation in codon 58.

These extensive differences have been identified within a rather small genetic region, the 792 bp upstream and 683 bp downstream of the *Pvdhfr-ts *coding region. To examine a somewhat more distant locus, a highly polymorphic repeat motif comprised of (TTAAA)_1 _(TCAAA)_n_(TTAAA)_n _(TGAAA)_n _that follows a string of 25 adenines in the TIGR reference sequence at nucleotide position -2608 upstream of the *Pvdhfr *coding region was assessed. The sequence of this region from a subset of isolates (data not shown) was determined. Figure 3 shows the 5' repeat motif and upstream, downstream and *dhfr *genotypes for isolates with the triple or quadruple mutant *dhfr *alleles, as well as for isolates in which recombination appears to have occurred. Colors in the *dhfr *genotype column reflect *dhfr *genotype, as in Figure 1. Colors upstream and downstream of *dhfr *reflect similarity of flanking regions. A variety of 5' repeat types were found, and the degree of recombination occurring between the 5' repeat region and the *dhfr *gene was examined. For example, it was found that substantial recombination upstream of *dhfr *has occurred among the isolates from Vanuatu; to a lesser extent recombination upstream of *dhfr *is seen among the isolates from PNG. Recombination has occurred upstream of *dhfr *in one Thai isolate bearing the 57I/58R/61M/117T allele (row 2). This isolate shares the 5' repeat motif with Thai isolates bearing the 58R/117N allele (row 1). There is one unusual Indonesian quadruple mutant allele whose 5' repeat motif is unlike those from the other Indonesian quadruple mutants (row 9), and is more similar to the 5' repeat motifs seen in isolates from Vanuatu (row 10). The 5' repeat motif carried by the Indonesian quadruple mutants differed from that seen in the Thai quadruple mutants, and these in turn differed from the repeat motifs carried by the PNG and Vanuatu triple and quadruple mutants. In conclusion, this additional sequencing of the 5' repeat motif bolsters the finding that highly mutant *Pvdhfr *alleles have arisen multiple times. Not only are differences in the *dhfr *coding region observed, but differences in the upstream and downstream flanking regions, and in the 5' repeat, are evidenced as well.

**Figure 4:**
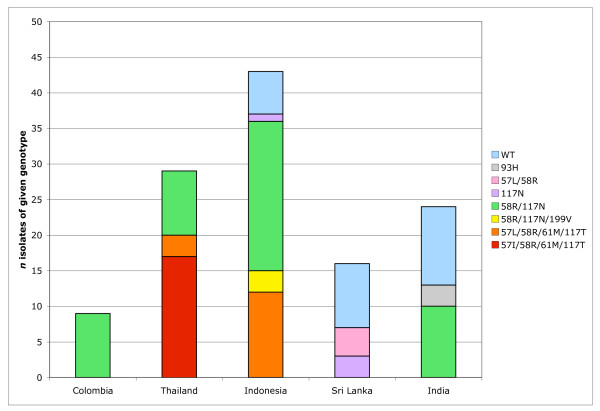
**Distribution of *P. vivax dhfr *alleles by region**. Note that indels are not considered. Isolates from Papua New Guinea and Vanuatu are excluded because they were included in this study specifically because they carry highly mutant *dhfr *alleles; isolates obtained from MR4 were also excluded.

The prevalence of mutations in *P. vivax dhfr *was also assessed by geographic region (Figure 4). Isolates from Papua New Guinea and Vanuatu are not included in this figure because they were part of this study specifically because they carried highly mutant *dhfr*. The four isolates obtained through MR4 are also not included. In the present sample set, triple and quadruple mutant *dhfr *alleles were identified only in isolates from Papua Indonesia, Papua New Guinea, Vanuatu and the Thai/Myanmar border.

In conclusion, highly pyrimethamine-resistant *Pvdhfr *alleles appear to have arisen three times, with origins in Thailand, Indonesia, and PNG/Vanuatu, and their diversity is dramatically higher than that seen in *P. falciparum*. The contrast in these parameters suggests that drug resistance may arise and spread very differently in the two species.

The isolates included in the present study came principally from Asia. Within this relatively small number of samples, three distinct origins of alleles bearing the 117 threonine mutation that is associated, in concert with mutations in other codons, with very high resistance to pyrimethamine were identified (for review see [8]). Moreover, isolates that express the same amino acid sequence of some very common double mutant alleles differ in the codons that encode the residue. Presumably this reflects very strong convergent evolution of the resistant enzyme. In contrast, in *P. falciparum *the two common double mutant *dhfr *alleles have arisen a few times, but the nucleotide sequences that encode the amino acid changes at residue 51 or 59 are the same. Most striking, the highly pyrimethamine-resistant triple mutant allele has arisen only rarely in *P. falciparum *[13,16]. Thus, the very wide selective sweep of a few resistant alleles that characterizes *Pfdhfr *does not seem to be the pattern for *Pvdhfr*.

What factors could explain the discrepancy between the frequency with which highly mutant alleles arise in *Pfdhfr *versus *Pvdhfr*? First, it is important to note that the paucity of origins of highly mutant *Pfdhfr *was unexpected. Given the intrinsic mutation rate of approximately 1.6 × 10^-4^/locus/generation [27], and assuming that roughly 10^8 ^to 10^12 ^parasites may reside within a single patient, mutations in *dhfr *would be expected to arise *de novo *in every patient. For example, a study combining *in vitro *culture of *P. falciparum *under pyrimethamine pressure and mathematical modeling found a mutation rate of 2.5 × 10^-9 ^mutations/*dhfr *gene/replication, though they assessed only two codons for non-synonymous substitutions [28].

It may be possible that differences in the frequency with which resistance-conferring mutations arise and spread in *Pvdhfr *versus *Pfdhfr *are due to differences in the mutation rates of the parasite species. However, it is not currently possible to assess this hypothesis, as experimentally determined *P. vivax *mutation rate and recombination rate are unknown. This study highlights that *P. vivax *and *P. falciparum *are different in important ways, notably that the origin and spread of drug resistance may not occur in the same manner in these two species. In addition, as sulphadoxine-pyrimetamine is not generally used intentionally to combat *P. vivax *parasites, the level of drug pressure exerted against parasites of this species is likely quite variable. This variable drug pressure may have an impact on the speed with which resistance-conferring mutations in *P. vivax dhfr *arise and are propagated through the population.

Other work has outlined important differences in the basic biology of *P. vivax *and *P. falciparum*. For example, *P. vivax *has a latent hypnozoite stage in the liver, in which relapses may occur months or even years following the primary infection; *P. falciparum *does not have this characteristic. In addition, gametocytogenesis is quite different in the two species, with the *P. falciparum *sexual cycle delayed with respect to the asexual cycle. In addition, *P. vivax *gametocytes at all stages of development are susceptible to drugs that kill asexual stage parasites, while only *P. falciparum *stage 1–3 gametocytes are susceptible to most antimalarials (for review see [29]). It is important to conduct additional studies to understand the biological basis for the finding that drug resistance-conferring mutations in *dhfr *have arisen and spread in a different manner in *P. vivax *versus *P. falciparum*.

In the present study quadruple mutant *P. vivax dhfr *alleles were assessed from Indonesia, Thailand and PNG. Mutant *dhfr *alleles containing the highly pyrimethamine-resistance conferring 117 threonine mutation were assessed from Vanuatu. Two alleles were identified: 58R/61M/117T and 57L/61M/117T/173F, and these alleles cluster with the quadruple mutant alleles from Papua New Guinea. Thus, the highly mutant *dhfr *alleles found in this study (those including the 117T mutation in concert with mutations in other codons) have arisen three times, with origins in Thailand, Indonesia, and PNG/Vanuatu. It will be of great future interest to assess the haplotyes of highly mutant *P. vivax *alleles from additional locales in order to determine the global diversity of these alleles. This study reveals a heavy antifolate selection on the *P. vivax *population even though the drugs have not been recommended for the treatment of infections of this species. Thus, the results also reflect the very high exposure of *P. vivax *to sulphadoxine-pyrimethamine, and reveal a major factor contributing to the development and spread of drug resistance.

In conclusion, the present study describes the origin and dissemination of highly mutant *P. vivax dhfr *alleles. Importantly, the pattern of selective sweep generally evidenced in *P. falciparum *was not observed; rather, independent mutations are common. Understanding the means by which mutations in *P. vivax dhfr *arise and spread may have important implications for malaria control policy [30]. Further, it is important to understand gene flow among *P. vivax *populations in order to predict how resistance to other drugs, for which molecular markers do not yet exist, may spread.

## Authors' contributions

VNH developed the experimental protocol, participated in amplification, sequencing and data analysis, and drafted the manuscript. AA participated in amplification, sequencing and data analysis. SKP participated in amplification and sample acquisition. KR, HCH, AM, MTO, QC, HJ and KN-B participated in sample acquisition and study coordination. CHS conceived of the study and participated in its design and coordination, and helped to draft the manuscript. All authors read and approved the final manuscript.
